# Big Data on Climatic and Environmental Parameters Associated with Acute Ocular Surface Symptoms and Therapeutic Assessment: Eye Drops Sales, Google Trends and Environmental Changes

**DOI:** 10.3390/vision9040096

**Published:** 2025-11-28

**Authors:** Felipe Barbosa Galvão Azzem Ferraz, Mateus Maia Marzola, Marina Zilio Fantucci, Adriana de Andrade Batista Murashima, Beatriz Carneiro Cintra, Denny Marcos Garcia, Eduardo Melani Rocha

**Affiliations:** Core of Research in Ocular Physiopathology and Therapeutics (NAP-FTO), Department of Ophthalmology, Otorhinolaryngology and Head & Neck Surgery, Ribeirão Preto Medical School, University of São Paulo, Ribeirao Preto CEP 14049-900, SP, Brazil; marinazf@fmrp.usp.br (M.Z.F.); adrimurashima@fmrp.usp.br (A.d.A.B.M.); beatrizcintra@usp.br (B.C.C.); dmgarcia@fmrp.usp.br (D.M.G.)

**Keywords:** dry eye, ocular surface, eye drops, climate, environment, Google

## Abstract

Ocular surface (OS) and dry eye (DE) symptoms are frequent ophthalmic complaints influenced by climate and pollution related with acute and chronic ocular surface symptoms. This study assessed their association with environmental conditions in São Paulo metropolitan area (2016–2020), including air temperature, humidity, atmospheric pressure, ozone (O_3_), particulate matter (PM), using IQVIA eye drop sales data and Google search trends. Sympathomimetic decongestant sales correlated with higher temperature (r = 0.434, *p* = 0.0021), UV radiation (r = 0.643, *p* < 0.0001), and ozone (r = 0.491, *p* = 0.0004). Artificial tears and lubricants correlated with ozone (r = 0.452, *p* = 0.0012) and with searches for “red eye” (r = 0.505, *p* = 0.0005) and “stye” (r = 0.599, *p* < 0.0001). To address multicollinearity, Principal Component Analysis (PCA) was applied, with the first two components (PC1 and PC2) explaining 87.3% of variance. Regression models using these components were significant for decongestant sales and “stye” searches. Eye drop sales and search trends thus emerge as potential indicators of OS and DE symptoms, reflecting environmental conditions and informing prevention strategies.

## 1. Introduction

Dry eye symptoms significantly impairs daily activities such as reading, driving, and computer use [1,2]. It can progress to Dry Eye Disease (DED), which affects 3% to 50% of the global population, with prevalence increasing with age and sex [3,4,5]. The wide variation in prevalence reflects differences in study populations and diagnostic criteria. Although rising incidence has been linked to environmental factors, including climate and air pollution indices measured at outdoor monitoring stations, the underlying mechanisms remain poorly understood and are difficult to establish with current diagnostic tools [6,7,8,9,10].

The absence of reliable biomarkers and the limited accuracy of existing diagnostic tests further hinder detection. Clinical manifestations range from mild discomfort to, in severe cases, corneal and ocular surface damage that may compromise vision, making it difficult to estimate DE symptoms prevalence outside hospital settings and to identify microgeographic and environmental influences on the ocular surface [10,11,12,13,14,15].

Environmental and climatic changes in recent decades have been associated with increased incidence of multiple diseases [16,17,18,19,20,21,22]. However, DE frequently lacks a clearly defined cause and, in most cases, has no definitive cure [23,24].

In this study, we investigated the relationship between dry eye symptoms and environmental conditions, hypothesizing that external environmental variables associated with dry eye symptoms correlate with increased sales of eye drops for symptom relief and with online searches related to ocular surface discomfort. If confirmed, such associations would support the use of environmental monitoring to anticipate fluctuations in DE symptoms prevalence and to identify potential triggers, thereby informing preventive strategies [25,26,27,28,29,30,31]. Given that the ocular surface is among the tissues most sensitive to environmental changes, it may also serve as an indicator of such variations [32,33].

Following approaches used to detect other population-level health phenomena, such as seasonal patterns in disease incidence, we analyzed the temporal frequency of Google searches related to DE symptoms to identify potential trends [34,35,36,37,38]. We further evaluated whether these variations correspond with sales of ocular surface eye drops across the same regions and periods, as previously hypothesized by our group [39]. Finally, the study aimed to examine the relationship between climatic and environmental variations in the metropolitan region of São Paulo, Brazil, online search activity related to DED, and eye drop sales within the state of São Paulo.

## 2. Methodology

This retrospective cross-sectional study is based on climate data, sales records, and Google search trends. The accessed databases cover the period between March 2016 and February 2020. The environmental outdoor parameters were obtained from the Environmental Company of the State of São Paulo (Companhia Ambiental do Estado de São Paulo—CETESB, available at: https://cetesb.sp.gov.br, accessed on 28 April 2020.), covering the metropolitan region of São Paulo, SP, Brazil. The parameters include Fine inhalable particles (PM_2.5_), Inhalable particles (PM_10_), Ozone (O_3_), atmospheric pressure (AP), ultraviolet solar radiation (UVR), air temperature (T ºC), and relative humidity (RH).

We could not obtain climate and pollution data from the whole São Paulo state and the entire analysis period. The reasons were discontinuity of data collection in some monitoring stations and vast areas where the climate and environment data are not monitored. We used the mean values of reliable data from the most populated areas to get around this limitation. This limitation meant that while the pollution and climate data fall within the region and period covered by the eye drop sales and Google search data described below, they do not fully coincide.

IQVIA, an American multinational company that conducts market research for the pharmaceutical industry, provided data on eye drop sales, among other services. For this study, we analyzed two therapeutic classes: Sympathomimetic Ophthalmic Decongestants and Artificial Tears/Ocular Lubricants. Therefore, our data source for these sales parameters was secondary, and we were unable to isolate data specifically for the metropolitan region used in the environmental analysis.

Regarding Google searches, we used the Google Trends tool, which allows querying how frequently a term was searched in a specific region over a given period (available at: http://google.com/trends/, accessed on 28 April 2020). In this study, we analyzed the terms “coceira no olho” (itchy eye), “olho seco” (dry eye), “olho vermelho” (red eye), and “terçol” (stye), searched within the state of São Paulo between 31 July 2016, and 28 February 2020. The values range from 0 to 100, where 100 represents the peak popularity of a search term, and 50 indicates that the term was half as popular at that time. Google does not provide search data at the city level or exclusively for the metropolitan region monthly.

### Statistical Method

We allocated the data based on monthly occurrences for each year and calculated the monthly averages over the four years. The mean, standard deviation, median, and range were calculated for each parameter related to climate, pollution, sales of eye drops in the Decongestant and Ocular Lubricant classes, and symptom searches on Google Trends.

The Pearson correlation test was applied to assess the relationship between the relative number of eye drop bottles sold and each environmental parameter in the metropolitan region of São Paulo. The same analysis was conducted between Google search results and each environmental parameter, as well as between the relative number of eye drop bottles sold and Google search results. The following r values were used to interpret correlation strength: 0.1 to 0.39 as weak, 0.4 to 0.69 as moderate, and above 0.7 as strong [40]. The cross-correlation test was used to identify potential time lags (up to −3 months) between the studied variables.

Principal Component Analysis (PCA) was employed to reduce the dimensionality of the environmental dataset, which comprised several correlated variables, by transforming them into a smaller set of uncorrelated principal components. The principal components explaining the highest proportion of variance were retained for further analysis. Multiple regression models were then constructed using these principal components as predictors to assess their combined and individual effects on the response variables of interest. This approach not only addresses potential multicollinearity among the environmental variables but also enhances the interpretability and reliability of the regression analysis.

All analyses were performed using R software version 4.1.1 (R Foundation for Statistical Computing, Vienna, Austria). A significance threshold of α = 0.05 was applied for all statistical tests.

## 3. Results

Table 1 presents values of climate, eyedrop sales, and symptoms variables distributed per month. Each analyzed parameter’s mean, standard deviation, median and range were obtained from March 2016 to February 2020. Table 2 is a correlation matrix among all variable series, compared in pairs using Pearson’s Correlation Coefficient. Among the most relevant findings, we highlight the positive correlations between decongestant eye drop sales and higher temperature (r = 0.434, *p* = 0.0021), greater UV radiation incidence (r = 0.643, *p* < 0.0001), and higher ozone concentration (r = 0.491, *p* = 0.0004). Additionally, increased lubricant sales correlated positively with ozone concentration (r = 0.452, *p* = 0.0012). Google search trends for the terms stye and red eye also showed positive correlations with lubricant sales (r = 0.505, *p* = 0.0005 and r = 0.599, *p* < 0.0001, respectively).

**Figure 1:**
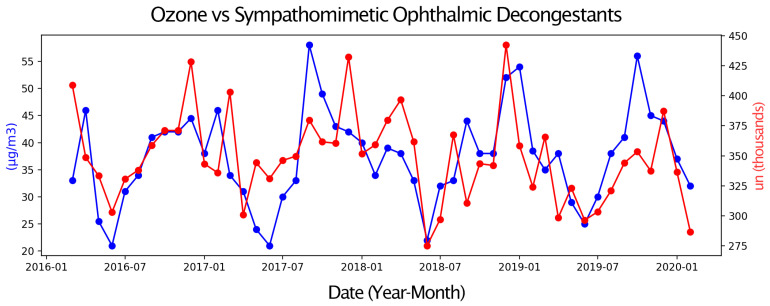
Graph showing the temporal overlap between the sales series of Sympathomimetic Ophthalmic Decongestants (in thousands of units) in the State of São Paulo and Ozone concentration (in µg/m^3^) in the Metropolitan Region of São Paulo, SP, from 1 March 2016 to 28 February 2020.

**Figure 2:**
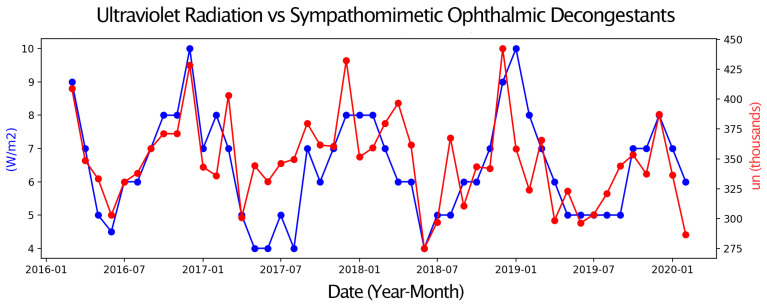
Graph showing the temporal overlap between the sales series of Sympathomimetic Ophthalmic Decongestants (in thousands of units) in the State of São Paulo and Ultraviolet Radiation (in W/m^2^) in the Metropolitan Region of São Paulo, SP, from 1 March 2016 to 28 February 2020.

**Figure 3:**
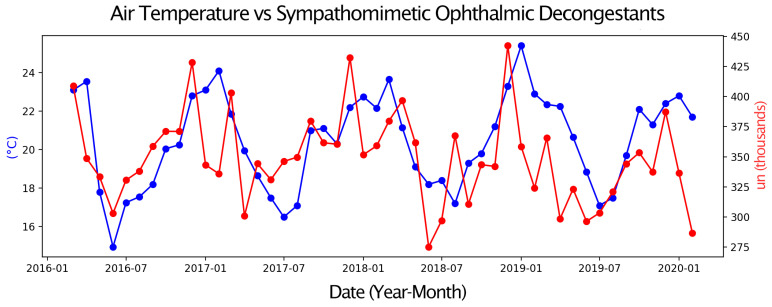
Graph showing the temporal overlap between the sales series of Sympathomimetic Ophthalmic Decongestants (in thousands of units) in the State of São Paulo and Air Temperature (in °C) in the Metropolitan Region of São Paulo, SP, from 1 March 2016 to 28 February 2020.

**Figure 4:**
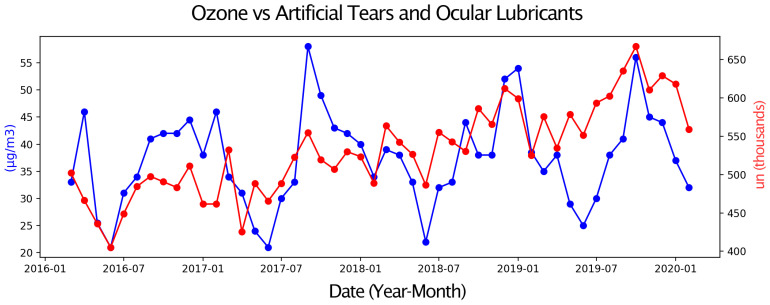
Graph showing the temporal overlap between the sales series of Artificial Tears and Ocular Lubricants (in thousands of units) in the State of São Paulo and Ozone Concentration (in µg/m^3^) in the Metropolitan Region of São Paulo, SP, from 1 March 2016 to 28 February 2020.

**Figure 5:**
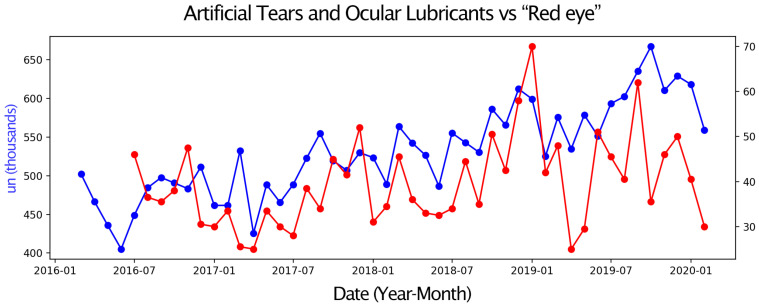
Graph showing the temporal overlap between the sales series of Artificial Tears and Ocular Lubricants (in thousands of units) in the State of São Paulo (blue) and the search term “red eye” (search volume normalized on a scale from 0 to 100) in the State of São Paulo from (red) 1 March 2016 to 28 February 2020.

**Figure 6:**
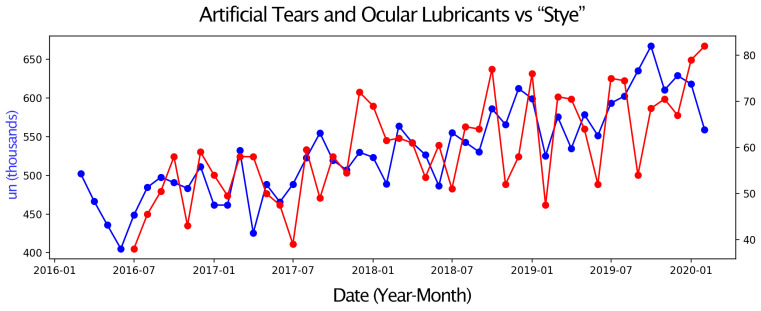
Graph showing the temporal overlap between the sales series of Artificial Tears and Ocular Lubricants (in thousands of units) in the State of São Paulo (blue) and the search term “stye” (search volume normalized on a scale from 0 to 100) in the State of São Paulo from (red) 1 March 2016 to 28 February 2020.

Next, pairs of time series were compared, with one representing eye drop sales and the other an environmental parameter or a Google Trends search term. Only graphs with moderate or strong correlations are presented. These graphs display two overlapping time series, allowing for a visual assessment of their relationship over the four-year study period (Figure 1, Figure 2, Figure 3, Figure 4, Figure 5 and Figure 6).

Sales of Sympathomimetic Ophthalmic Decongestants increased with higher ozone concentrations (Figure 1) and also with increased ultraviolet radiation (Figure 2) and air temperature (Figure 3). Sales of Artificial tears and Ocular Lubricant eye drops increased as ozone levels rose (Figure 4). Additionally, higher search volumes for “red eye” (Figure 5) and “stye” (Figure 6) showed a positive correlation with the sales of these eye drops.

We generated the cross-correlation matrix shown in Table 3 using Pearson’s correlation coefficient. As previously explained, this test was used to identify potential time lags (up to −3 months) between the studied variables. Moderate correlations are highlighted in bold. Some values showed stronger correlations when looking at three months back. For example, the correlation coefficient between the sales of Sympathomimetic Ophthalmic Decongestants and relative humidity was −0.138 with no time lag. However, when considering humidity from three months earlier, the impact on sales was greater, −0.344.

**Figure 7:**
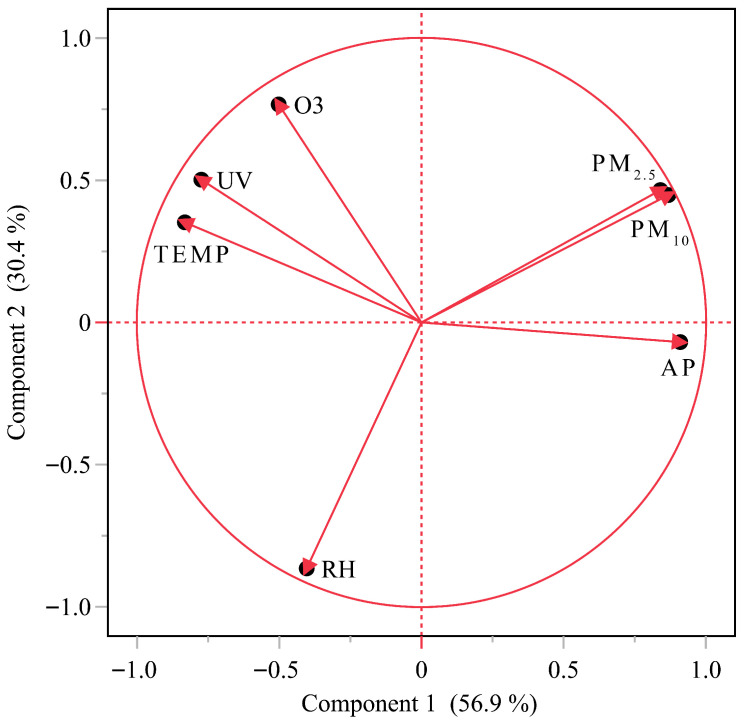
Correlation circle of the principal component analysis (PCA). The plot displays the relationships among environmental variables (O_3_, UV, TEMP, RH, PM_2.5_, and AP) on the first two principal components, which explain 56.9% and 30.4% of the total variance, respectively.

Figure 7 illustrates the relationships between the original variables and the principal components (PCs). The variance explained by the first two components was 56.9% and 30.4%, respectively. PC1 captures the contrast between PM_2.5_, PM_10_, and AP, which are positively correlated, and O_3_, UV, and TEMP, which are negatively correlated. PC2 highlights the negative correlation with RH. The loadings of the first two components are presented in Table 4.

The results of the multiple regression analysis assessing the relationships between the response variables and the first two principal components are presented in Table 5. The regression models for Descon and “Stye” were statistically significant, with both PC1 and PC2 emerging as significant predictors. For Lubrif, the model demonstrated marginal significance, with PC2 acting as a significant positive predictor, while PC1 was not significant. In contrast, the regression models for “Itchy eye,” “Dry eye,” and “Red eye” were not statistically significant and exhibited very low explanatory power.

## 4. Discussion

This study analyzes the relationship between environmental parameters and the sales of symptomatic eye drops for ocular discomfort, based on the hypothesis that worsening environmental conditions increase the demand for these eye drops, while improvements reduce sales. Additionally, we investigated the connection between these environmental factors, eye drop sales, and related search terms on Google.

We observed that higher ozone concentrations were associated with increased sales of both classes of eye drops, suggesting that DE symptoms worsen with elevated ozone levels [41]. This effect may be due to an inflammatory response and even reduced tear production directly caused by this environmental factor [7,42,43].

Temperature also influences eye drop consumption, with higher temperatures associated with increased sales. This may be explained by temperature fluctuations that worsen DE symptoms [6,29,31]. The same pattern is observed with ultraviolet radiation: higher levels lead to increased ocular discomfort [6], which explains the observed rise in sales of Sympathomimetic Ophthalmic Decongestants.

Low relative humidity affects the tear film [6,25] and worsens DE symptoms [30,38], as suggested by previous studies. However, we found only a very weak correlation between humidity and sales of both eye drop classes. When analyzing data from three months prior using cross-correlation, humidity showed a greater influence on Decongestant sales, but the correlation remained weak. Future studies with shorter time intervals, such as daily variations, may reveal stronger correlations and further support this hypothesis.

It is important to note that the excessive and prolonged use of eye drops can lead to ocular surface discomfort that mimics the clinical manifestations of dry eye disease (DED), but also may induce toxic and allergic reactions, or predispose to OS infection. This effect is attributed mainly to the preservatives commonly present in these formulations, which may disrupt tear film stability, increase osmolarity, and compromise epithelial integrity, triggering toxic innate immune reaction, hypersensibility, and predisposing to infection or, in preservative-free presentation, induce contamination [44].

Another study identified atmospheric pressure as a factor influencing the occurrence of DE symptoms [45]. However, the correlations found between pressure and symptomatic eye drop sales were weak. Only the Decongestant approached the threshold for a moderate correlation. This suggests some level of influence, but it is either minimal or was not detected due to the monthly time scale, the range of variation, geographic discrepancies, or other methodological limitations of our study.

We found weak correlations for inhalable (PM_10_) and fine inhalable particles (PM_2.5_), with studies showing mixed results. Some found no link to DED [45], while others reported associations only for PM_2.5_ [41,46,47] or both [42]. Differences may stem from methodological limitations or the complex composition of these pollutants, which include carbon, sulfates, heavy metals, and hydrocarbons, making their effects on DED difficult to determine.

Only the search terms stye (hordeolum) and red eye showed a moderate correlation with Artificial tears and Lubricant sales. Previous studies have used Google searches to track conjunctivitis outbreaks [34], predict seasonal influenza [35], and assess population mental health dynamics [36]. While these studies suggest that search trends can anticipate disease outbreaks, the selected terms or study population may not have strongly correlated with environmental changes or caused discomfort at a detectable frequency or intensity.

A study on DED used Google Trends as an epidemiological tool, analyzing U.S. macro-regions and linking search terms to environmental factors like temperature, humidity, and air quality index, with findings similar to ours [38].

However, to our knowledge, this is the first study to explore DED in relation to search trends, eye drop sales, and a wider range of environmental factors, especially in the Global South.

This study focuses on a region of São Paulo state and has limitations. It does not account for other measures people might take for eye discomfort or confirm that increased sales were due to discomfort, dry eye, or ocular surface disease. Since DED is often chronic, immediate responses, such as searching for information or purchasing eye drops, may be less likely than with acute illnesses. Because of that, we tested the time lag and found some correlations. Additionally, using monthly intervals and only considering outdoor environmental factors prevents detecting short-term correlations or responses to indoor conditions, such as air-conditioned spaces. Also, the demographic, socio-economic, ethnic, psychological, genetic, and medical backgrounds of the users providing data by Google Trends remain largely unknown. Moreover, this analysis was not performed on patients formally diagnosed with DED but at the population level, which introduces potential confounders and limits the ability to establish direct causal associations.

The results indicate a moderate correlation between ozone levels, air temperature, and UV radiation with the sales of Decongestant Eye Drops. There was also a moderate correlation between ozone levels and the sales of Artificial Tears and Lubricants. Additionally, sales of this eye drop class were moderately correlated with Google searches for “stye” and “red eye”. All other correlations between environmental factors, eye drop sales, and search terms were weak.

These findings suggest that DE is influenced by climate and pollution. Sales of symptomatic eye drops reflect changes in environmental conditions and could serve as an indirect monitoring tool. Similarly, Google searches for DE-related symptoms align with eye drop sales and environmental variations. In the future, monitoring eyedrop sales and internet searches for ocular symptoms may help identify climate changes and DE symptoms incidence in epidemiological studies in big population cities and remote geographic regions.

## Figures and Tables

**Table 1:** Descriptive statistics for each collected parameter, including mean, standard deviation, median, and range.

Variable		JAN	FEB	MAR	APR	MAY	JUN	JUL	AUG	SEP	OCT	NOV	DEC
PM_2.5_ μg/m^3^	Mean	13.1	12.4	13.5	17.0	17.4	20.5	24.6	18.9	21.0	14.5	12.6	14.3
	SD	2.4	2.4	1.7	4.9	1.4	1.7	4.9	2.8	6.1	2.4	0.5	1.3
	Median	12.5	12.0	13.5	17.0	17.3	20.5	23.3	19.0	18.5	14.5	12.8	14.0
	Range	5.5	5.5	4.0	12.0	3.0	3.0	11.0	5.0	11.0	3.0	5.0	10.0
PM_10_ μg/m^3^	Mean	20.4	20.1	22.1	28.4	27.8	34.5	41.3	32.4	33.7	24.3	20.6	23.2
	SD	2.4	3.9	2.3	7.2	2.9	4.0	4.0	4.5	10.0	4.0	1.5	1.5
	Median	20.0	19.3	22.5	27.5	27.0	34.0	41.0	32.0	30.0	24.0	20.0	23.0
	Range 5.0	9.0	4.5	17.5	6.0	7.0	17.0	10.0	22.0	8.5	3.0	3.5	
Ozone μg/m^3^	Mean	42.3	37.6	35.3	38.3	47.9	42.9	33.3	20.4	8.4	46.3	42.9	45.6
	SD	7.9	6.2	6.1	4.0	10.0	9.0	2.4	8.1	1.9	9.2	7.2	9.9
	Median	39.0	36.3	34.5	38.0	47.3	41.5	30.5	33.5	43.5	45.5	42.5	44.3
	Range	17.0	14.0	14.0	13.0	30.0	25.0	5.0	30.0	4.0	34.0	30.0	30.0
Atmospheric Pressure (kPa)	Mean	922.9	923.0	924.1	925.0	926.0	926.8	929.1	927.9	926.4	933.7	930.0	922.4
	SD	1.2	0.3	1.1	1.2	1.5	0.9	0.6	0.5	0.6	0.5	0.6	0.6
	Median	922.8	923.0	924.3	925.9	926.1	928.4	927.7	926.0	926.4	933.8	929.8	922.8
	Range	2.9	0.7	2.4	2.5	2.4	1.5	4.3	4.1	3.1	1.0	1.3	1.4
Ultraviolet Radiation (W/m^2^)	Mean	8.0	7.5	7.3	8.0	7.8	5.0	3.0	5.0	6.3	6.8	7.8	7.4
	SD	1.4	1.0	1.0	0.8	0.5	0.5	0.8	0.5	0.8	0.7	0.9	0.5
	Median	7.5	8.0	7.0	6.0	7.5	5.0	5.0	6.5	6.5	6.5	7.0	8.5
	Range	4.0	2.0	3.0	2.0	2.0	1.5	1.0	2.0	3.0	3.0	2.0	1.0
Air Temperature (°C)	Mean	23.5	22.7	22.7	21.7	19.1	17.4	17.3	19.6	20.5	20.8	20.8	22.7
	SD	1.3	1.0	0.8	1.5	1.7	1.0	0.7	1.4	1.2	1.3	1.1	0.7
	Median	23.0	22.5	22.7	21.7	17.9	17.4	17.9	19.5	20.6	20.8	20.8	22.6
	Range	2.6	2.4	1.8	3.6	2.8	3.9	1.9	0.4	2.8	2.3	1.1	1.1
Relative Humidity (%)	Mean	76.3	77.0	79.0	75.3	78.0	76.6	68.9	72.9	71.1	74.6	76.0	73.0
	SD	2.5	4.6	1.8	4.6	4.1	2.9	3.2	2.2	5.7	4.6	1.1	4.2
	Median	76.5	76.5	79.3	75.5	78.8	76.3	68.8	73.3	73.3	73.8	76.3	72.5
	Range	6.0	11.0	3.5	10.0	9.5	6.0	7.0	5.0	12.0	11.0	2.5	9.0
Decon (bottle units)	Mean	347.5	326.4	389.3	336.1	340.6	301.4	319.3	344.1	348.3	357.4	352.7	422.6
	SD	9.7	30.3	20.2	46.5	16.5	23.0	23.1	19.6	29.1	11.8	15.7	24.2
	Median	347.5	329.9	391.4	324.7	338.8	299.7	317.0	343.9	351.4	357.5	351.2	430.4
	Range	22.1	72.7	43.1	98.1	38.4	55.8	49.1	46.7	69.1	27.8	33.5	54.9
Lubric (bottle units)	Mean	550.5	508.7	543.3	492.2	507.3	477.1	521.4	538.0	554.4	565.7	541.5	570.4
	SD	72.0	42.5	33.0	56.0	60.3	60.3	65.0	49.2	58.6	78.4	57.4	58.6
	Median	561.2	507.2	548.0	500.5	507.4	476.0	521.8	532.6	542.5	552.7	536.2	570.9
	Range	156.4	97.4	73.4	116.6	142.7	146.1	144.4	117.8	137.4	176.2	126.9	117.5
Itchy eye	Mean	20.5	10.6	9.5	3.2	15.5	6.0	14.8	13.8	11.4	19.6	16.3	16.1
	SD	15.9	10.2	9.5	5.5	13.8	10.4	9.9	8.2	8.3	8.5	13.3	10.9
	Median	21.8	9.0	9.5	0.0	20.0	0.0	19.0	10.0	13.8	20.5	18.3	20.5
	Range	38.5	24.5	19.0	9.5	26.5	18.0	21.0	17.0	18.0	20.5	28.5	23.5
Dry eye	Mean	27.9	22.6	32.7	19.5	41.3	27.0	15.0	37.4	25.0	28.0	27.9	32.8
	SD	10.5	6.3	10.1	7.9	5.8	5.1	11.9	9.2	11.2	11.9	9.1	20.7
	Median	29.3	23.8	34.0	16.0	40.5	28.0	15.5	39.5	23.5	26.5	30.3	31.0
	Range	21.0	15.0	20.0	14.5	11.5	10.0	29.0	21.5	27.0	27.0	21.0	39.0
Red eye	Mean	42.9	35.0	39.7	28.7	32.0	37.8	38.4	40.0	41.6	42.3	44.4	47.6
	SD	18.7	5.0	12.3	6.4	2.2	11.5	8.9	3.4	13.6	6.8	2.8	11.9
	Median	35.8	34.0	45.5	25.0	33.0	33.5	39.8	39.5	35.3	41.5	44.3	51.0
	Range	40.0	12.0	22.5	11.0	4.0	21.0	18.0	8.0	28.0	15.0	6.0	27.5
Stye	Mean	69.5	60.1	63.7	63.2	55.8	53.3	50.8	61.0	54.4	65.4	55.0	64.0
	SD	11.2	15.8	6.7	6.5	7.3	6.6	17.2	12.1	6.8	9.2	11.5	6.7
	Median	72.5	55.5	62.0	61.0	53.5	52.0	45.0	62.0	52.3	63.3	53.3	63.0
	Range	25.0	34.5	13.0	12.5	14.0	13.0	37.0	29.0	15.0	19.0	27.5	14.0

Abbreviations: PM_2.5_—Fine Inhalable Particles in µg/m^3^; PM_10_—Inhalable Particles in µg/m^3^; Ozone in µg/m^3^; Atmospheric Pressure in hPa; Ultraviolet Radiation in W/m^2^; Air Temperature in °C; Relative Humidity in %; Decon—Sympathomimetic Ophthalmic Decongestant expressed in thousands of bottles; Lubric—Artificial Tears and Ocular Lubricant expressed in thousands of bottles; SD—Standard deviation. Google search terms (“itchy eye,” “dry eye,” “red eye,” and “stye”) were normalized by Google on a scale from 0 to 100.

**Table 2:** Correlation matrix among all variable series.

	Temperature	RH	AP	UV	O_3_	PM_10_	PM_2.5_	Decon	Lubric	Itchy Eye	Dry Eye	Red Eye	Stye
**Temp**	1.000												
*p*-value	–												
**RH**	0.103	**1.000**											
*p*-value	0.49	–											
**AP**	**−0.768**	−0.304	**1.000**										
*p*-value	<0.0001 *	0.0356	–										
**UV**	**0.787**	−0.145	**−0.678**	**1.000**									
*p*-value	<0.0001 *	0.32	<0.0001 *	–									
**O_3_**	**0.622**	**−0.435**	**−0.477**	**0.680**	**1.000**								
*p*-value	<0.0001 *	0.0020 *	0.0006 *	<0.0001 *	–								
**PM_10_**	**−0.502**	**−0.694**	**0.716**	**−0.458**	−0.125	**1.000**							
*p*-value	0.0003 *	<0.0001 *	<0.0001 *	0.0011 *	0.40	–							
**PM_2.5_**	**−0.460**	**−0.679**	**0.677**	**−0.427**	−0.103	**0.983**	**1.000**						
*p*-value	0.0010 *	<0.0001 *	<0.0001 *	0.0024 *	0.48	<0.0001 *	–						
**Decon**	**0.434**	−0.138	−0.381	**0.643**	**0.491**	−0.292	−0.264	**1.000**					
*p*-value	0.0021 *	0.35	0.0075	<0.0001 *	0.0004 *	0.0442	0.07	–					
**Lubric**	0.313	−0.118	−0.130	0.183	**0.452**	−0.064	−0.053	0.160	**1.000**				
*p*-value	0.0304	0.42	0.38	0.21	0.0012 *	0.67	0.72	0.28	—				
**“Itchy eye”**	0.051	0.169	−0.200	0.117	0.012	−0.196	−0.183	−0.111	0.373	**1.000**			
*p*-value	0.74	0.27	0.19	0.45	0.94	0.20	0.24	0.47	0.0126 *	–			
**“Dry eye”**	0.064	0.188	−0.080	−0.034	0.034	−0.181	−0.159	0.049	0.339	0.275	**1.000**		
*p*-value	0.68	0.22	0.60	0.83	0.83	0.24	0.30	0.75	0.0245 *	0.07 *	–		
**“Red eye”**	0.189	−0.103	−0.129	0.303	0.359	−0.058	−0.000	0.274	**0.505**	0.236	0.012	**1.000**	
*p*-value	0.22	0.51	0.40	0.0459 *	0.0166 *	0.71	0.99	0.07	0.0005 *	0.12	0.94	–	
**“Stye”**	0.350	0.359	−0.306	0.136	0.170	−0.391	−0.383	−0.047	**0.599**	**0.458**	0.390	0.193	**1.000**
*p*-value	0.0198 *	0.0166 *	0.0432 *	0.38	0.27	0.0087 *	0.0102 *	0.76	<0.0001 *	0.0018 *	0.0089 *	0.21	–

Abbreviations: RH—Relative Humidity; AP—Atmospheric Pressure; O_3_—Ozone; UV—Ultraviolet Radiation; PM_10_—Inhalable Particles; PM_2.5_—Fine Inhalable Particles; Decon—Sympathomimetic Ophthalmic Decongestant; Lubric—Artificial Tears and Ocular Lubricant. The values in bold indicate at least a moderate correlation between the two series. “*” indicates statistically significant correlations (*p* < 0.05).

**Table 3:** Cross-correlation matrix between all environmental parameters, Google search terms, and eye drop categories.

	Temperature	RH	AP	UV	O_3_	PM_10_	PM_2.5_
**Decon**	**0.483 [0]**	−0.344 [−3]	−0.381 [0]	**0.643 [0]**	**0.491 [0]**		
*p*-value	**0.0021**	0.007	0.0026	**<0.001**	**<0.001**	ns	ns
**Lubric**	0.313 [0]				**0.452 [0]**		
*p*-value	0.0149	ns	ns	ns	**0.0003**	ns	ns
“**Itchy eye**”		−0.325 [−3]				0.340 [−3]	0.363 [−3]
*p*-value	ns	0.0112	ns	ns	ns	0.0078	0.0043
**“Dry eye”**							
*p*-value	ns	ns	ns	ns	ns	ns	ns
**“Red eye”**	−0.363 [−3]		0.329 [−3]	−0.332 [−3]	0.359 [0]	0.340 [−3]	0.374 [−3]
*p*-value	0.0044	ns	0.0104	0.0097	0.0166	0.0079	0.0033
**“Stye”**	0.350 [0]	0.359 [0]	−0.306 [0]		0.359 [0]	−0.391 [0]	−0.384 [0]
*p*-value	0.0061	0.0048	0.0173	ns	0.0087	0.002	0.0025

Abbreviations: RH—Relative Humidity; AP—Atmospheric Pressure; UV—Ultraviolet Radiation; O_3_—Ozone; PM_10_—Inhalable Particles; PM_2.5_—Fine Inhalable Particles; Decon—Sympathomimetic Ophthalmic Decongestant; Lubric—Artificial Tears and Ocular Lubricant; ns—not significant. The values in bold indicate at least a moderate correlation between the two series.

**Table 4:** Loadings of environmental variables on the first two Principal Components Identified by Principal Component Analysis (PCA).

Variable	PC1	PC2
PM25	0.84	0.47
PM_10_	0.87	0.45
O_3_	−0.50	0.77
AP	0.91	−0.07
UV	−0.77	0.50
TEMP	−0.83	0.35
RH	−0.40	−0.86

**Table 5:** Results of multiple regression analysis relating response variables to two principal components.

Variable	Intercept Coefficient	PC1 Coefficient	PC2 Coefficient	Model F Value	R-Squared
	(*p*-Value)	(*p*-Value)	(*p*-Value)	(*p*-Value)	
Decon	348.8 (<0.0001)	−8.8 (0.0003)	8.9 (0.0060)	11.7 (<0.0001)	0.34
Lubrif	530.8 (<0.0001)	−5.9 (0.16)	11.4 (0.0476)	3.10 (0.0548)	0.12
“Itchy eye”	13.5 (<0.0001)	−0.89 (0.27)	−0.75 (0.52)	0.89 (0.42)	0.04
“Dry eye”	27.9 (<0.0001)	−0.66 (0.46)	−1.1 (0.39)	0.678 (0.51)	0.03
“Red eye”	29.5 (<0.0001)	−0.90 (0.22)	1.9 (0.07)	2.41 (0.10)	0.11
“Stye”	59.70 (<0.0001)	−2.0 (0.0112)	−1.1 (0.32)	4.20 (0.0219)	0.17

## Data Availability

The original contributions presented in this study are included in this article. Further inquiries can be directed to the corresponding authors.

## Abbreviations

The following abbreviations are used in this manuscript:

OSD	Ocular surface diseases
DED	Directory of open access journals
O_3_	Ozone
PM	Particulate matter

UVR	Ultraviolet solar radiation
OS	Ocular surface
PM_2.5_	Fine inhalable particles
PM_10_	Inhalable particles
AP	Atmospheric pressure
RH	Relative humidity
